# Crosstalk between NFκB-dependent astrocytic CXCL1 and neuron CXCR2 plays a role in descending pain facilitation

**DOI:** 10.1186/s12974-018-1391-2

**Published:** 2019-01-03

**Authors:** Huadong Ni, Yungong Wang, Kang An, Qianying Liu, Longsheng Xu, Chunyan Zhu, Housheng Deng, Qiuli He, Tingting Wang, Miao Xu, Ying Zheng, Bing Huang, Jianqiao Fang, Ming Yao

**Affiliations:** 1 Department of Anesthesiology and Pain Research center, The First Affiliated Hospital of Jiaxing University, Jiaxing, 314001 China; 2 Zhejiang Chinese Medicine University, Hangzhou, 310053 China; 3 Department of Anesthesiology, Zhuzhou Central Hospital, Zhuzhou, 412000 China; 4 Department of Anesthesiology, Affiliated Drum Tower Hospital of Medical School of Nanjing University, Nanjing, 210004 China

**Keywords:** CXCL1, CXCR2, Periaqueductal gray, Glia-neuron interaction, Bone cancer pain, NFκB

## Abstract

**Background:**

Despite accumulating evidence on the role of glial cells and their associated chemicals in mechanisms of pain, few studies have addressed the potential role of chemokines in the descending facilitation of chronic pain. We aimed to study the hypothesis that CXCL1/CXCR2 axis in the periaqueductal gray (PAG), a co-restructure of the descending nociceptive system, is involved in descending pain facilitation.

**Methods:**

Intramedullary injection of Walker 256 mammary gland carcinoma cells of adult female Sprague Dawley rats was used to establish a bone cancer pain (BCP) model. RT-PCR, Western blot, and immunohistochemistry were performed to detect p*Nfkb*, *Cxcl1*, and *Cxcr2* and their protein expression in the ventrolateral PAG (vlPAG). Immunohistochemical co-staining with NeuN, GFAP, and CD11 were used to examine the cellular location of pNFκB, CXCL1, and CXCR2. The effects of NFκB and CXCR2 antagonists and CXCL1 neutralizing antibody on pain hypersensitivity were evaluated by behavioral testing.

**Results:**

BCP induced cortical bone damage and persistent mechanical allodynia and increased the expression of pNFκB, CXCL1, and CXCR2 in vlPAG. The induced phosphorylation of NFκB was co-localized with GFAP and NeuN, but not with CD11. Micro-injection of BAY11-7082 attenuated BCP and reduced CXCL1 increase in the spinal cord. The expression level of CXCL1 in vlPAG showed co-localization with GFAP, but not with CD11 and NeuN. Micro-administration of CXCL1 neutralizing antibody from 6 to 9 days after inoculation attenuated mechanical allodynia. Furthermore, vlPAG application of CXCL1 elicited pain hypersensitivity in normal rats. Interestingly, CXCR2 was upregulated in vlPAG neurons (not with CD11 and GFAP) after BCP. CXCR2 antagonist SB225002 completely blocked the CXCL1-induced mechanical allodynia and attenuated BCP-induced pain hypersensitivity.

**Conclusion:**

The NFκB-dependent CXCL1-CXCR2 signaling cascade played a role in glial-neuron interactions and in descending facilitation of BCP.

**Electronic supplementary material:**

The online version of this article (10.1186/s12974-018-1391-2) contains supplementary material, which is available to authorized users.

## Background

Bone cancer pain (BCP) is a severe and chronic pain that has a negative impact on the quality of life of cancer patients. Advancements in the mechanisms and pharmacotherapy of BCP have achieved limited success, and commonly-used analgesics have resulted in little or no response. Therefore, novel and more efficacious therapies are urgently needed for improving the patients’ quality of life.

Recent studies have demonstrated that hyperalgesia in animal models with persistent pain is closely associated with the activation of “top-down” modulatory circuits involving descending facilitation or descending inhibition [1, 2]. The ventrolateral periaqueductal gray (vlPAG) is a substantial component of the descending pain modulatory network and exerts inhibitory or excitatory control on pain transmission via the rostral ventromedial medulla (RVM), which in turn projects to the spinal dorsal horn [3–5]. The increased net descending pain modulatory drive leads to an amplification of the pain [2, 6–8]. However, the cellular and molecular mechanisms underlying the injury-induced synaptic plasticity in the descending facilitation of vlPAG circuitry are poorly understood.

Accumulating evidence demonstrated an important role of neuroimmune interactions in chronic pain [9]. Glial hyperactivity and its associated chemokines contribute to persistent pain in the spinal dorsal horn [9, 10]. Recent studies on the models of chronic pain have demonstrated glial activation in PAG [11, 12] associated with changes in cytokines/chemokines [13]. Moreover, the chemokine CXCL1, also known as keratinocyte-derived chemokines (KC) or growth-related oncogene (GRO), is a member of CXC family and has been demonstrated to play a critical role in the induction and maintenance of inflammatory pain [14], neuropathic pain [15, 16], and BCP [17] facilitation via its preferred receptor, CXCR2 [18, 19]. These data suggested that CXCL1 and CXCR2 are involved in astroglial-neuronal interaction in the spinal cord under chronic pain conditions. Similar processes that are involved in the alteration of descending pain modulation may occur in vlPAG.

Nuclear factor kappa B (NFκB) is a transcription factor that transduces extracellular signals to affect gene expression [17]. NFκB is involved in TNFα-induced CXCL1 expression in primary astrocytes [20]. Moreover, emerging evidences have indicated that the activation of NFκB following inflammatory pain, neuropathic pain, and BCP is related to the generation of chronic pain [21–23]. Studies have reported that NFκB mediates CXCL1 expression in the spinal astrocytes and contributes to BCP [17]. Whether NFκB mediates CXCL1 expression in vlPAG astrocytes and contributes to the descending facilitation of BCP needs further investigation.

Hence, the present study was aimed to investigate the hypothesis that CXCL1/CXCR2 signaling in vlPAG is involved in descending the pain facilitation. We examined the expression and distribution of NFκB and CXCL1 in vlPAG after inoculation of Walker 256 cells. We also evaluated the role of NFκB in CXCL1 production at vlPAG and pain hypersensitivity after BCP. We further investigated the expression and distribution of CXCR2 in vlPAG and the antinociceptive effect of CXCR2 antagonist. The release of CXCL1 from astrocytes mediated by NFκB and the subsequent activation of its CXCR2 receptor on neurons may present astroglial-neuronal interaction that is involved in the descending facilitation of BCP-like behavior in rats.

## Methods

### Animal preparation

Female Sprague Dawley rats (180–200 g) were housed in plastic cages and maintained on a 12-h light/dark cycle at 22–25 °C with food and water available. All efforts were made to minimize the animal suffering and the number of animals used. All experimental procedures received prior approval from the Animal Use and Care Committee for Research and Education of the Jiaxing University (Jiaxing, China) and the ethical guidelines to investigate experimental pain in conscious animals [24].

### Experimental protocol

**Fig. 1:**
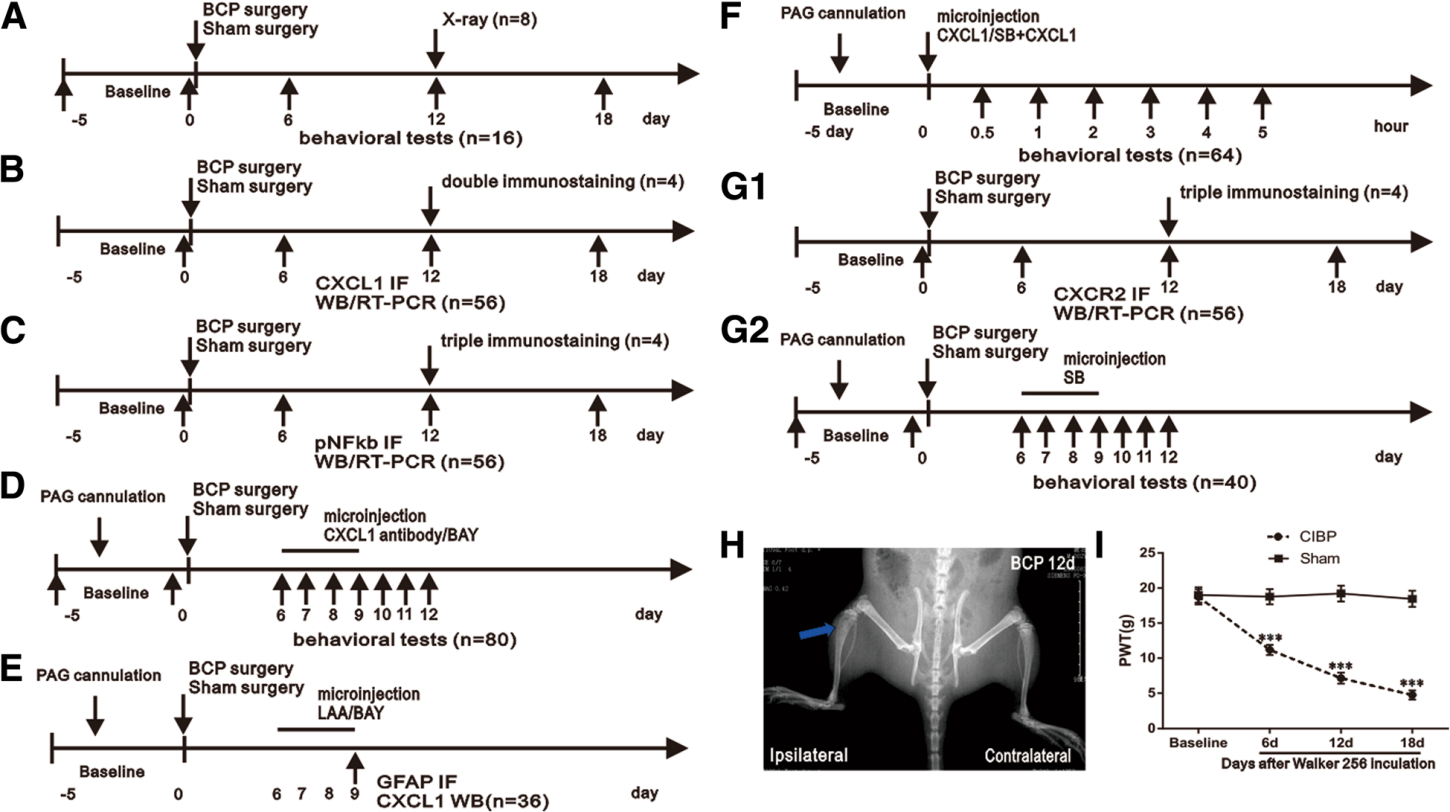
BCP induced mechanical allodynia of hindpaw. **a**–**g2** Experimental paradigms. **h** Radiography showing cortical bone damage in the tibial bone (arrow) at 12 days after inoculation. **i** BCP showing prominent decrease in the PWTs of ipsilateral hind paw on POD 6 to 18. *n* = 8 per group, ***p < 0.001 vs. sham group. LAA, L-α- aminoadipic acid, BAY, BAY11-7082, SB, SB225002

The experimental protocol was divided into seven parts (total rat = 424):

1. Establish and validate a bone cancer pain model (total rat = 24). Sixteen rats were randomly divided into BCP and sham (injection heat-killed Walker 256 cell) groups (*n* = 8). PWT of the ipsilateral hind paw was observed 1 day before and 6, 12, and 18 days after surgery. Additional 8 rats were then randomly divided into two groups: sham and 12 days after surgery (*n* = 4). These rats were then radiographed (Fig. 1a).
2. Determine whether the CXCL1 upregulated occurs in the vlPAG and the cell distribution of CXCL1 in vlPAG following BCP (total rat = 60). Fifty-six rats were randomly divided into seven groups: naïve, BCP (6, 12, 18 days) group, and sham (6, 12, 18 days) groups (*n* = 8). After each time point, rats were anesthetized and then perfused with fixative or decapitated for immunohistochemistry (*n* = 4) or Western blot/RT-PCR analysis (*n* = 4). Tissue sections from additional four rats of day-12 surgery animals were labeled with a fluorescent marker for CXCL1 and co-labeled with CD11 (microglial marker), GFAP (astrocytic marker), or NeuN (neuronal marker) (Fig. 1b).
3. Determine whether the NFκB activation occurs in the vlPAG and the cell distribution of p-NFκB in vlPAG following BCP (total rat = 60). Fifty-six rats were randomly divided into seven groups: naïve, BCP (6, 12, 18 days) group, sham (6, 12, 18 days) groups (*n* = 8). After each time point, rats were anesthetized and then perfused with fixative or decapitated for immunohistochemistry (*n* = 4) or Western blot/RT-PCR analysis (*n* = 4). Tissue sections from additional four rats of day-12 surgery animals were labeled with a fluorescent marker for p-NFκB and co-labeled with CD11, GFAP, or NeuN (Fig. 1c).
4. Determine whether CXCL1 neutralizing antibody and NFκB inhibitor attenuates mechanical allodynia caused by BCP (total rat = 80). Eighty rats were randomly divided into ten groups: sham + serum group (*n* = 8), BCP + serum group (*n* = 8), BCP + CXCL1 neutralizing antibody 8 μg group (*n* = 8), BCP + CXCL1 neutralizing antibody 4 μg group (*n* = 8) groups, sham + CXCL1 neutralizing antibody 8 μg group (*n* = 8), sham + veh (PBS) group (*n* = 8), BCP + veh group (*n* = 8), BCP + BAY 10 μg group (*n* = 8), BCP + BAY 1 μg group (*n* = 8) groups, and sham + BAY 10 μg group (*n* = 8). The rats continuously received a vlPAG microinjection of above drugs once daily for four consecutive days (from day 6 to 9 after BCP), and we then detected the analgesic effect of CXCL1 and pNFκB inhibition 1 h later after micro-injection on postoperative days (POD) 6, 7, 8, 9, 10, 11, and 12, respectively (Fig. 1d).
5. Examining whether the BCP induces CXCL1 upregulation in vlPAG astrocytes via the NFκB pathway (total rat = 36). Twenty-four rats were randomly divided into three groups: sham + veh (saline) (*n* = 8), BCP + veh (*n* = 8), and BCP + LAA (*n* = 8). After repeated drug administration from days 6 to 9 after BCP (once daily for four consecutive days), rats were decapitated for CXCL1 Western blot analysis of the vlPAG (*n* = 4) and perfused with fixative for immunohistochemistry for GFAP (*n* = 4). Additional 12 rats were then randomly divided into three groups: sham + veh (PBS) group (*n* = 4), BCP + veh group (*n* = 4), and BCP + BAY 10 μg group (*n* = 4). We blocked the NFκB pathway with BAY (once daily from day 6 to 9 after BCP) and then detected CXCL1 protein level after the last injection (Fig. 1e).
6. Determine whether CXCL1 was sufficient to induce pain and how it is involved in the modulation of pain hypersensitivity (total rat = 64). Twenty-four rats were randomly divided into three groups: PBS (*n* = 8), CXCL1 100 ng (*n* = 8), and CXCL1 10 ng (*n* = 8). The naive rats received a vlPAG microinjection of above drugs, and we then detected the analgesic effect of drugs 0.5, 1, 2, 3, 4 and 5 h later after micro-injection respectively. Additional 40 rats were then randomly divided into five groups: PBS + SB (SB225002) 20 μg group (*n* = 8), 5%DMSO + CXCL1 100 ng group (*n* = 8), SB 20 μg + CXCL1 100 ng group (*n* = 8), SB 5 μg + CXCL1 100 ng group, and PBS + 5%DMSO group (*n* = 8). The naive rats received a vlPAG microinjection of above drugs, and we then detected the analgesic effect of drugs 0.5, 1, 2, 3, 4 and 5 h later after micro-injection respectively (Fig. 1f).
7. Determine whether the CXCR2 upregulated occurs in the vlPAG and the cell distribution of CXCR2 in vlPAG following BCP (total rat = 100). Fifty-six rats were randomly divided into seven groups: naïve, BCP (6, 12, 18 days) group, and sham (6, 12, 18 days) groups (*n* = 8). After each time point, rats were anesthetized and then perfused with fixative or decapitated for immunohistochemistry (*n* = 4) or Western blot/RT-PCR analysis (*n* = 4). Another 40 rats were randomly divided into five groups: BCP + veh (5%DMSO) group (*n* = 8), BCP + SB 20 μg group (*n* = 8), BCP + SB 5 μg group (*n* = 8), sham + veh group (*n* = 8), and sham + SB 20 μg group (*n* = 8). The rats continuously received a vlPAG microinjection of above drugs once daily for four consecutive days (from day 6 to 9 after BCP), and we then detected the analgesic effect of CXCR2 inhibition 1 h later after micro-injection on postoperative days (POD) 6, 7, 8, 9, 10, 11, and 12, respectively. Tissue sections from additional four rats of day-12 surgery animals were labeled with a fluorescent marker for CXCR2 and co-labeled with CD11, GFAP, or NeuN (Fig. 1g1–g2).

### vlPAG cannulation and microinjections

Rats were anesthetized with isoflurane (4–5% induction, 1.5–2% maintenance) in a gas mixture of 25% O2/75% nitrogen and then placed in a stereotaxic instrument (RWD Life Science Co., China). A 7-mm stainless steel guide cannula (23-gauge) was implanted into the unilateral vlPAG (7.6 mm posterior to bregma, 0.6 mm lateral from the midline, and 5.8 mm beneath the surface of the skull) [25]. A dummy cannula (28-gauge stainless steel wire) was inserted into the guide cannula to reduce the incidence of infection and clogging. Rats were given at least 5 days to recover before experimentation. Drugs (0.5 μl) were administered through a 28-gauge injection cannula extending 0.5 mm beyond the tip of the guide cannula. The solution was infused with a pump at 0.1 μl/min for 5 min. Waiting for another 2 min, the injection cannula was gently removed.

The CXCL1 neutralizing antibody and control serum were from Sigma (St. Louis, USA) and diluted in sterile artificial cerebrospinal fluid (ACSF). The potent and selective antagonist of CXCR2 SB225002 was from Sigma (St. Louis, USA) and dissolved in 5% dimethylsulfoxide (DMSO). BAY11-7082, an NFκB inhibitor, was from Abcam (Cambridge, England) and dissolved in phosphate buffer saline (PBS). Exogenous CXCL1 was purchased from R&D Systems (Minneapolis, MN, USA) and dissolved in phosphate buffer saline (PBS). The astrocytic cytotoxin, L-α- aminoadipic acid (LAA), was from Sigma (St. Louis, USA) and dissolved in 0.9% saline. The injection cannula remained in place for an additional 2 min to prevent the backflow of the drug after completion of drug infusion. The microinjection of the drug was administered at 8 a.m. daily, which was 30 min before the behavioral test. The doses of CXCL1 neutralizing antibody (1 μg, 8 μg), SB225002 (1 μg, 10 μg), BAY11-7082 (1 μg, 4 μg, and 8 μg), CXCL1 (10 ng, 100 ng), and LAA (100 nmol) used in this experiment were based on our preliminary results and previous use in relevant studies [14, 17, 18]. The same volume of saline, 5%DMSO or PBS was injected as vehicle control. At the end of the experiment, the injection sites were histologically verified to be within the vlPAG (Additional file 1: Figure S1).

### Modeling of bone cancer pain rats

The BCP model has been established as previously described [26]. In brief, the animals were anesthetized by pentobarbital sodium (50 mg/kg, i.p.). A hole was carefully drilled into the lower one third of the right tibia for inoculation. Then, 10 μl of Walker 256 cells (2 × 10^5^ cells/ml) or heat-killed cells (sham group) were cautiously injected into the bone medullary canal. The cells were allowed to fill the bone cavity for 2 min. After that, the syringe was withdrawn and the injection site was immediately closed with bone wax.

### Radiology

To confirm cancer development in the tibia, rats were radiographed after 12 following implantation. After the rats were anesthetized with pentobarbital sodium, the right hind limbs were placed on X-ray film (Kodak, Italy) and exposed to X-ray for 1/20 s at 40 kVp. The images were taken from sham rats and BCP rats.

### Von Frey test for behavioral assessment

Mechanical allodynia of the right hind paw was measured with a von Frey monofilaments (BME-404; Institute of Biological Medicine, Academy of MedicalScience, Beijing, China) as described in our previous methods [27]. Animals were familiarized with the testing environment daily for at least 3 days before baseline testing. Rats were individually placed in transparent Plexiglas compartments (20 × 10 × 10 cm) for 30 min for habituation before each test. The values were averaged to yield a PWT after five consecutive tests at 10-s intervals. All the behavioral testing procedures were performed by researchers who were blinded to the group.

### Immunofluorescence

Immunofluorescence staining and double/ triple immunostaining were performed as previously described [12]. Briefly, the polyacrylamide hydrogel (PAG) tissues were harvested and cryosectioned at 35 μm. The sections were first incubated with 5% normal donkey serum in 0.1 M PBS for 2 h at room temperature. The sections were then incubated overnight at 4 °C with the following primary antibodies: CXCL1 antibody (1:100, rabbit; Boster), phosphor-NF-кB p65 (Ser536) (pNF-кB) antibody (Rabbit, 1:500; Sigma), CXCR2 antibody (1:100, rabbit; Abcam), GFAP antibody (1:1000, mouse; Sigma), CD11 antibody (1:250, mouse; Abcam), and NeuN antibody (1:300, mouse; Abcam). The sections were then incubated for 1 h with FITC- or Cy3-conjugated secondary antibodies (1500, Abcam) at room temperature. For double immunofluorescence, sections were incubated with a mixture of primary antibodies at 4 °C overnight, followed by a mixture of FITC- and Cy3-conjugated secondary antibodies. Sections were coverslipped using a water-based mounting medium containing DAPI (Bioss, China). The stained sections were examined using an Olympus fluorescence microscope, analyzed by Image Pro-plus 6.0 (Image Pro-Plus Kodak, USA), and images were taken with a CCD Spot camera. Negative controls in which the primary antibody was omitted or replaced with PBS were used to confirm immunospecificity.

### Real-time quantitative PCR

Total RNA was extracted from the vlPAG with Trizol reagent (Invitrogen, Carlsbad, CA). One microgram of total RNA was converted into cDNA using PrimeScriptRT reagent kit (Takara, Shiga, Japan). The cDNA was amplified using the following primers: CXCL1 forward,5′-GGCAGGGATTCACTTCAAGA-3′;CXCL1 reverse, 5′-ATCTTGAGCTCGGCAGTGTT -3′;pNF-кBforward,5′-TCTGCTTCCAGGTGACAGTG-3′;pNF-кB reverse, 5′-ATCTTGAGCTCGGCAGTGTT-3′;CXCR2 forward, 5′-CGTTCTGGTGACTTTGCTGA-3′;CXCR2 reverse,5′-ACAGAGCAGGTGCTTCGATT-3′; GAPDH forward, 5′-TCTCTGCTCCTCCCTGTTC-3′;GAPDH reverse, 5′-ACACCGACCTTCACCATCT-3′. The PCR reactions were performed in a real-time detection system (Rotor-Gene 6000, Corbett Research, Mortlake, Australia) by SYBR PremixEx Taq™ II kit (Takara). The reaction conditions consisted ofan initial denaturation step at 95 °C for 10 min, followed by 45 cycles at 95 °C for 5 s, 58 °C for 30 s, and 72 °C for 30 s. The data were analyzed using Rotor-Gene 6000 series software and evaluated using the Comparative CT method (2^− ΔΔCT^).

### Western blot

The vlPAG tissues were homogenized with ice-cold lysis buffer containing 150 mMNaCl, 20 mMTris-HCl (PH 7.6), 1 mM EDTA, 1% NP-40, 1 mM PMSF, protease inhibitor cocktail (Sigma, St. Louis, MO), and phosphatase inhibitor cocktail (Sigma, St. Louis, MO). Protein concentrations were determined by the BCA Protein assay (Pierce, Rockford, IL). Equal amounts of protein (30 μg) were loaded for each lane, separated by SDS-PAGE gel (10%), and transferred onto the nitrocellulose blots. After the transfer, the blots were first saturated by incubation in 10% skim milk for 1 h and then incubated overnight at 4 °C with antibody against CXCL1 (1:400, rabbit, Boster), CXCR2 (1:400, rabbit, Abcam), and p-NFkB p65 (Ser536) antibody (1:1000, rabbit, Sigma). For loading control, the blots were probed with GAPDH antibody (1:20000, mouse, Sigma). All the blots were further incubated overnight at 4 °C with primary antibody. Bound primary antibodies were detected following incubation at RT for 1 h with appropriate horseradish peroxidase-conjugated anti-rabbit or anti-mouse secondary antibody (1:10000, Jackson immunolab). Immunoreactive bands were detected by using enhanced chemiluminescence (Thermo Scientific) and exposed to X-ray films. GAPDH served as an internal control.

### Statistical analysis

SPSS version 20.0 was used to determine statistical significance. Results were expressed as mean ± SEM. Differences between the groups were compared using one-way ANOVA or two-way repeated measures or three-way repeated measures ANOVA followed by Newman-Keuls post hoc test. We used Shapiro-Wilk test to assess all the data distribution. As for the homogeneity analysis, we used the *F* test to test equality of variances in *t* test and Levene’s test of equality of error variances for ANOVA. *p* < 0.05 was considered to be statistically significant.

## Results

### Intramedullary inoculation of Walker 256 cells produces the destruction of rats’ tibiacortical bone and bone cancer pain

To verify the validation of BCP model, radiological imaging of rat tibia to assess the bone destruction 12 days after cancer cell inoculation was performed. Ipsilateral proximal epiphysis was disrupted in the bone marrow cavity 12 days post-inoculation (Fig. 1b), suggesting the development of bone cancer in the tibia. No radiological changes were found in the contralateral tibia (Fig. 1b) or control animals treated with heat-killed tumor cells. To further investigate the chronic pain status induced by BCP model, the mechanical allodynia of ipsilateral hind paws were evaluated (Fig. 1a). All rat groups showed no differences in the baseline hind paw withdrawal threshold (PWT) to mechanical stimulation (*p* > 0.05; Fig. 1c). However, the PWT of BCP hind paws were significantly lower than sham rats on day 6 (*F*_1,14_ = 1315, ***p < 0.001 vs. sham group; *n* = 8, two-way repeated measures ANOVA, Fig. 1c). With the progression of tumor, the PWT was gradually decreased in the inoculated hind paw from days 6 to 18 (*F*_1,14_ = 1315, ***p < 0.001 vs. sham group; *n* = 8, two-way repeated measures ANOVA, Fig. 1c).

### CXCL1 is persistently increased in vlPAG astrocytes after BCP

**Fig. 2:**
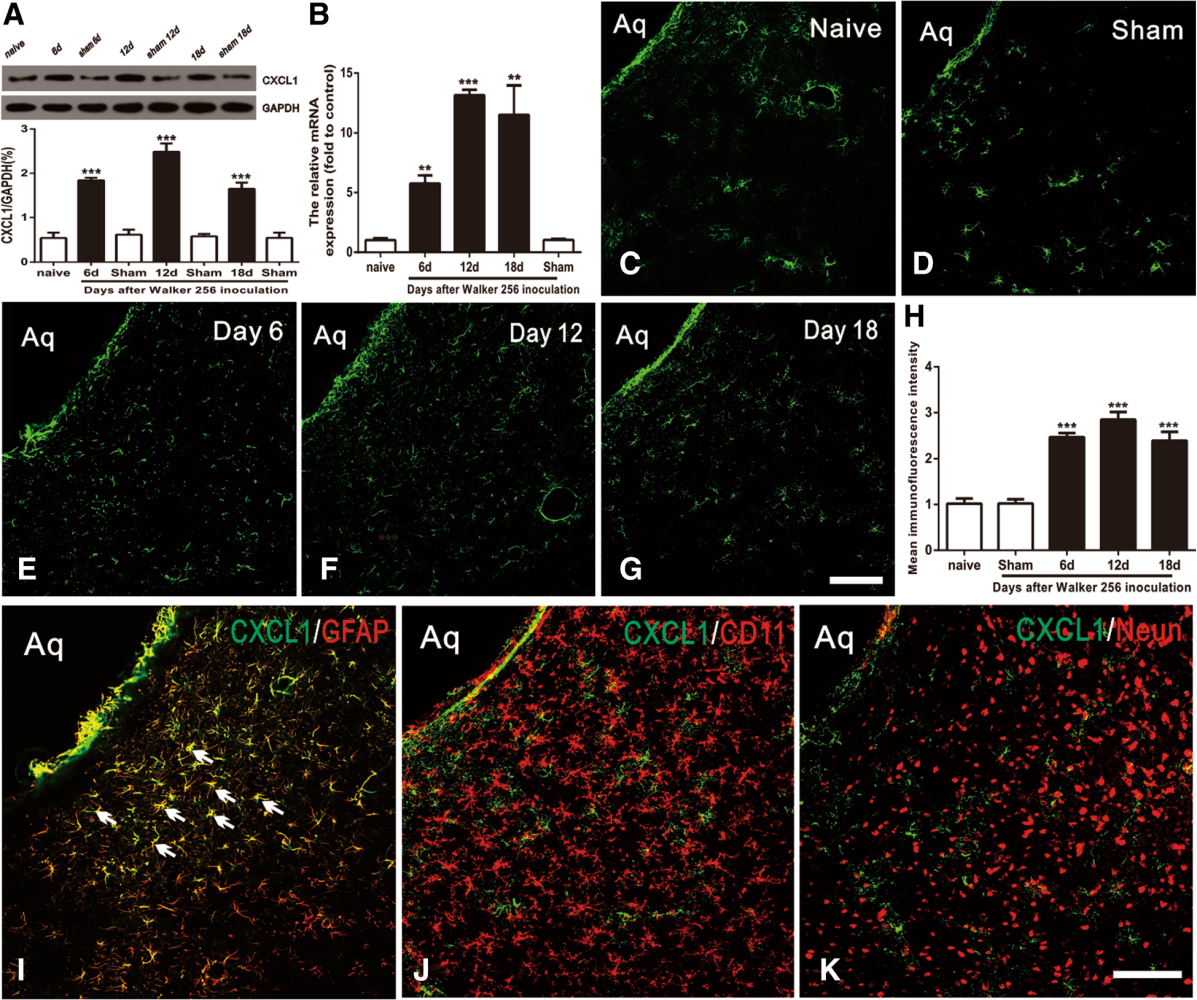
Expression and distribution of CXCL1 protein in vlPAG after BCP in rats. **a** Western blot analysis showing the time course of vlPAG CXCL1 expression in sham and BCP rats. CXCL1 protein upregulation was gradually increased from 6 days to 18 days. ***p < 0.001 vs. naïve group. **b** RT-PCR results showing the increase of CXCL1 mRNA expression in vlPAG after inoculation. CXCL1 mRNA upregulation was gradually increased from 6 days to 18 days. ***p < 0.001, **p < 0.01 vs. naïve control. **c**–**g** Immunostaining showing CXCL1-IR was increased in the spinal cord at 6 days (**e**), 12 days (**f**), and 18 days (**g**). **h** Statistical analysis of CXCL1-immunoreactive (IR) intensity further confirmed the increase of CXCL1 expression in vlPAG after BCP. ***p < 0.001 vs. naïve group. (**i**–**k**) Double staining showed CXCL1 was co-localized with astrocytic marker, GFAP (**i**), but not with microglial marker CD11 (**j**) or neuronal marker NeuN (**k**). *n* = 4. Scale bar: 100 um.Aq, aqueduct

Bone cancer-induced CXCL1 changes in the spinal cord are critical for the generation of BCP [17]. Here, we examined whether CXCL1/CXCR2 chemokine signaling in PAG could be functionally upregulated in the BCP state. We first detected the expression and distribution of CXCL1 in the vlPAG after BCP. Western blot analysis showed that BCP induced a rapid-onset and increased the expression of CXCL1 protein in the vlPAG from day 6 to 18 after BCP. The evident increase has begun on day 6, peaked by day 12, and reached at high level until day 18 (*F*_6,21_ = 175.4, ***p < 0.001 vs. naive group; *n* = 4, one way ANOVA, Fig. 2a). However, CXCL1 protein was at a low level in the vlPAG of sham-operated rats. These findings were additionally confirmed by RT-PCR. The results revealed a parallel and significant increase in the vlPAG CXCL1 mRNA at 6, 12, and 18 days in BCP animals (*F*_4,15_ = 96.21, ***p < 0.001, **p < 0.01 vs. naive group; *n* = 4, one way ANOVA, Fig. 2b). We then checked CXCL1 expression by immunostaining. Compared with the naive group and sham group (Fig. 2c, d), tumor cell inoculation induced a marked increase of CXCL1 expression in the vlPAG at days 6, 12, and 18 (Fig. 2e–g). Statistical analysis of CXCL1-immunoreactive (IR) intensity further confirmed the increase of CXCL1 expression in vlPAG after BCP (*F*_4,15_ = 159.8, ***p < 0.001 vs. naive group; *n* = 4, one way ANOVA, Fig. 2h).

To investigate the cell distribution of CXCL1 in vlPAG following BCP, we performed double immunostaining of CXCL1 at 12 days with three major nerve cell-specific markers: GFAP (for astrocytes), CD11 (for microglia), and NeuN (for neurons). Confocal images showed that CXCL1-IR was co-localized with the GFAP (Fig. 2i), but not with CD11 (Fig. 2j) or neuronal marker NeuN (Fig. 2k), suggesting that CXCL1 was induced by astrocytes, but not by neurons or microglia in BCP rats.

### NFκB activation in vlPAG astrocytes and neurons after Walker 256 cell inoculation

**Fig. 3:**
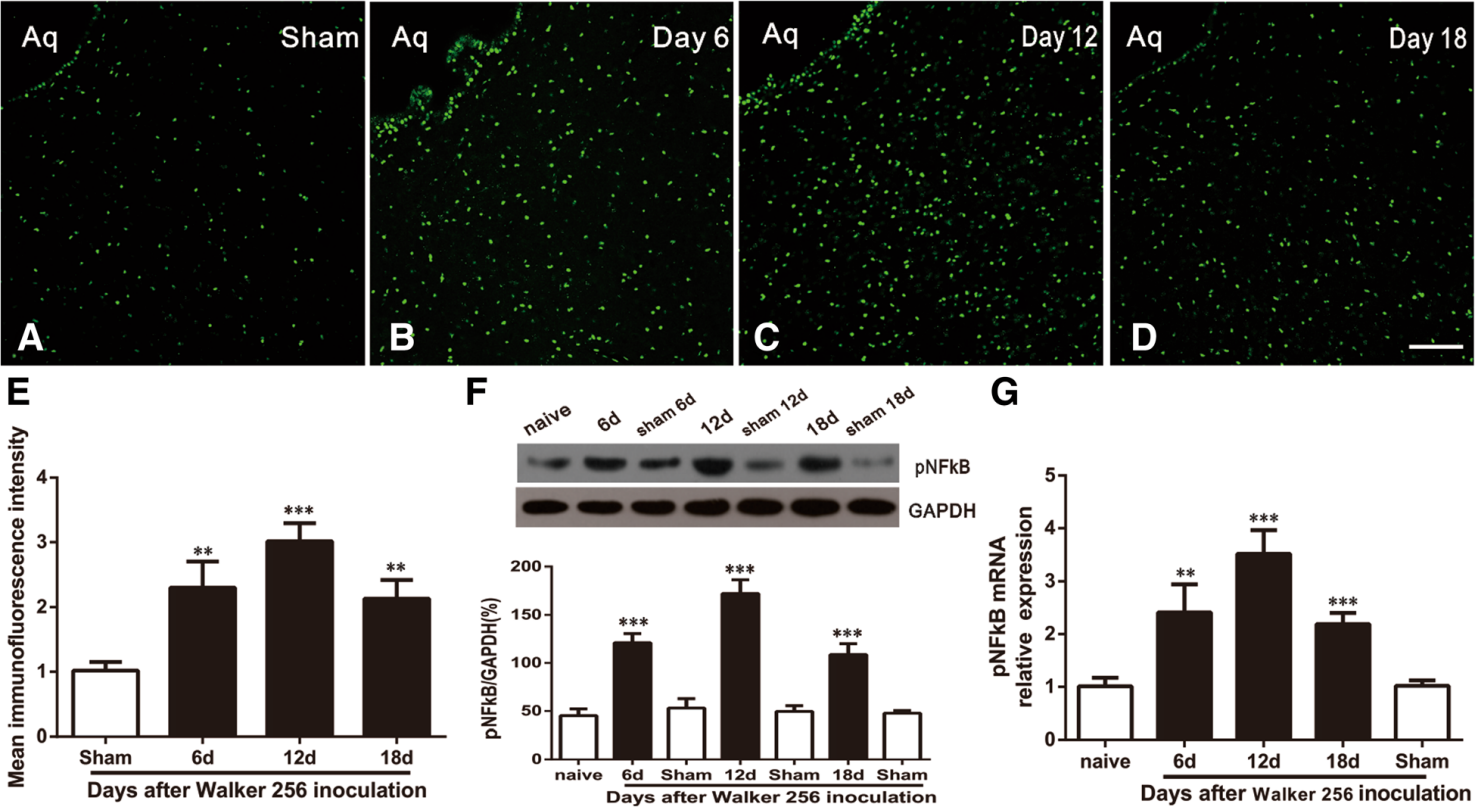
BCP induces time-dependent activation of NFκB in vlPAG. **a** Expression levels of pNFκB in the vlPAG in Sham group. **b**–**d** Upregulation of pNFκB in vlPAG on postoperative days (POD) 6, 12, and 18. **e** Statistical analysis of pNFκB intensity confirmed the increase of pNFκB expression in vlPAG after BCP. **f** Western blot results showed an increase of pNFκB in the vlPAG after tumor inoculation on days 6–18. **g** Real-time PCR further confirmed a parallel and significant increase in vlPAG at 6, 12, and 18 days in BCP. ***p < 0.001, **p < 0.01 vs. naive group or sham group. Four rats in each group. Scale bars: 100 μm in (**d**). Aq, aqueduct

NFκB is a ubiquitous transcription factor that control the expression of genes that are critical to both aggressive tumor growth and resistance to chemotherapy during cancer treatment [23, 28, 29]. To check whether NFκB pathway in vlPAG was involved in BCP hypersensitivity, we first evaluated NFκB activation after tumor cell inoculation. Immunostaining showed a low expression of phosphorylated NFκB (pNFκB) in sham-treated rats (Fig. 3a) and an increased expression in the BCP rats on days 6, 12, and 18 (Fig. 3b–d). Statistical analysis of pNFκB intensity confirmed the increase of pNFκB expression in vlPAG after BCP (*F*_3,12_ = 32.44, ***p < 0.001, **p < 0.01 vs. sham group; *n* = 4, one way ANOVA, Fig. 3e). Western blot and RT-PCR further showed that the pNFκB expression was gradually increased from day 6 to 18 (*F*_6,21_ = 111.1, ***p < 0.001 vs. naive group; *n* = 4, one way ANOVA, Fig. 3f) (*F*_4,15_ = 38.82, ***p < 0.001, **p < 0.01 vs. naive group; *n* = 4; Fig. 3g).

**Fig. 4:**
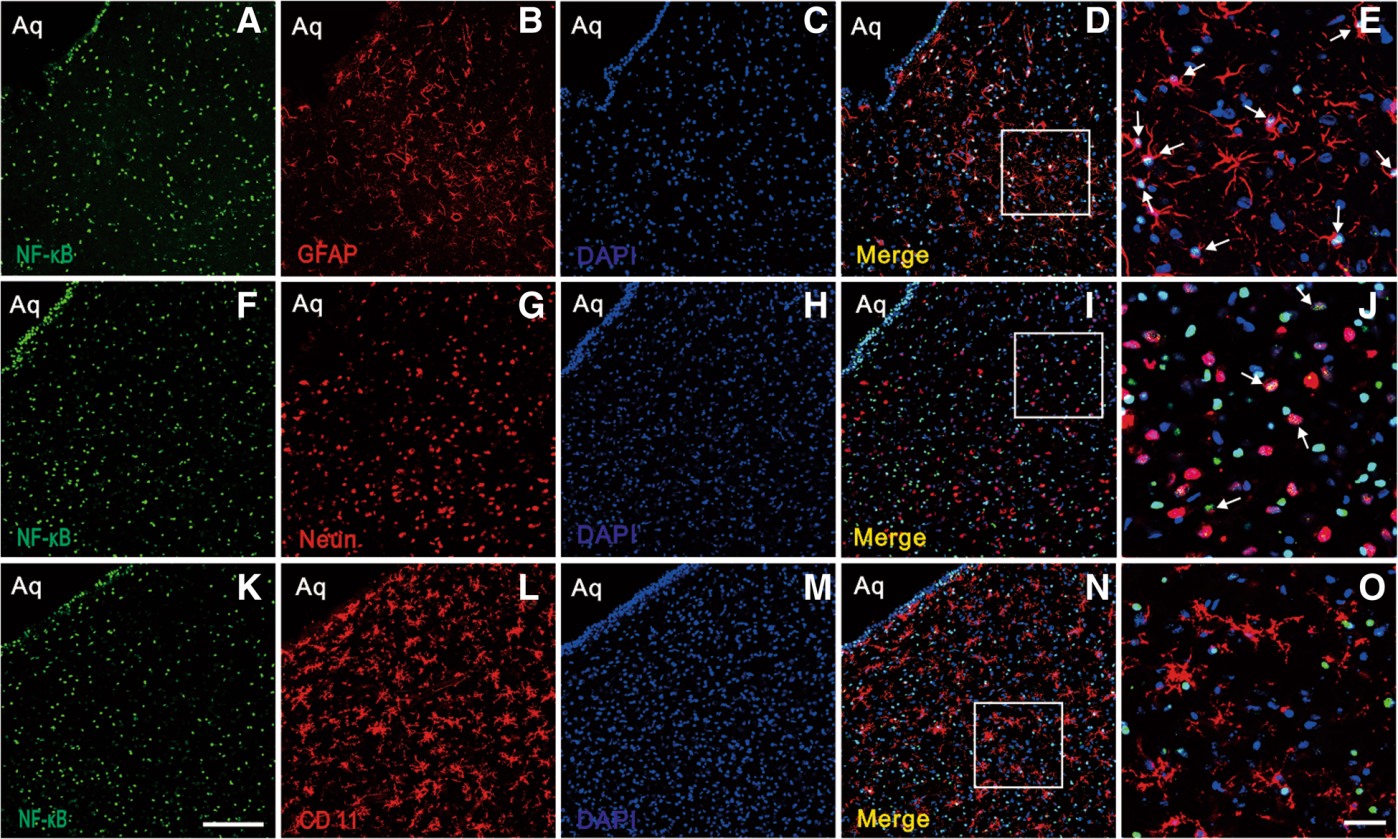
Cellular localization of pNFκB expression in vlPAG after BCP. Immunofluorescence triple staining of pNFκB (**a**, **f**, **k**), GFAP (**b**), Neun (**g**), CD11 (**l**), and DAPI (**c**, **h**, **m**) in vlPAG at day 12 after BCP showed that all pNFκB, glial cells, and neurons express DAPI + cells (**d**, **i**, **n**). Scale bar in (**k**): 100 μm. Immunofluorescence triple staining of high-magnification images were indicated in the white boxes of (**d**, **i**, **n**), and mainly co-localized with GFAP (**e**) and Neun (**j**), but not with microglia in BCP rats (**o**). Scale bar in (**o**): 50 μm.Aq, aqueduct. *n* = 4

To further define the cellular localization of pNFκB in vlPAG, triple staining of pNFκB with three different cell markers (Neun, GFAP, and CD11) and DAPI was performed. Results showed that pNFκB was increased in vlPAG on day 12 after BCP (Fig. 4a, f, k) and was mainly co-localized with GFAP (Fig. 4a–e) and Neun (Fig. 4f–j), but not with microglia in BCP rats (Fig. 4k–o).

### CXCL1 neutralizing antibody and NFκB inhibitor attenuates mechanical allodynia caused by BCP

**Fig. 5:**
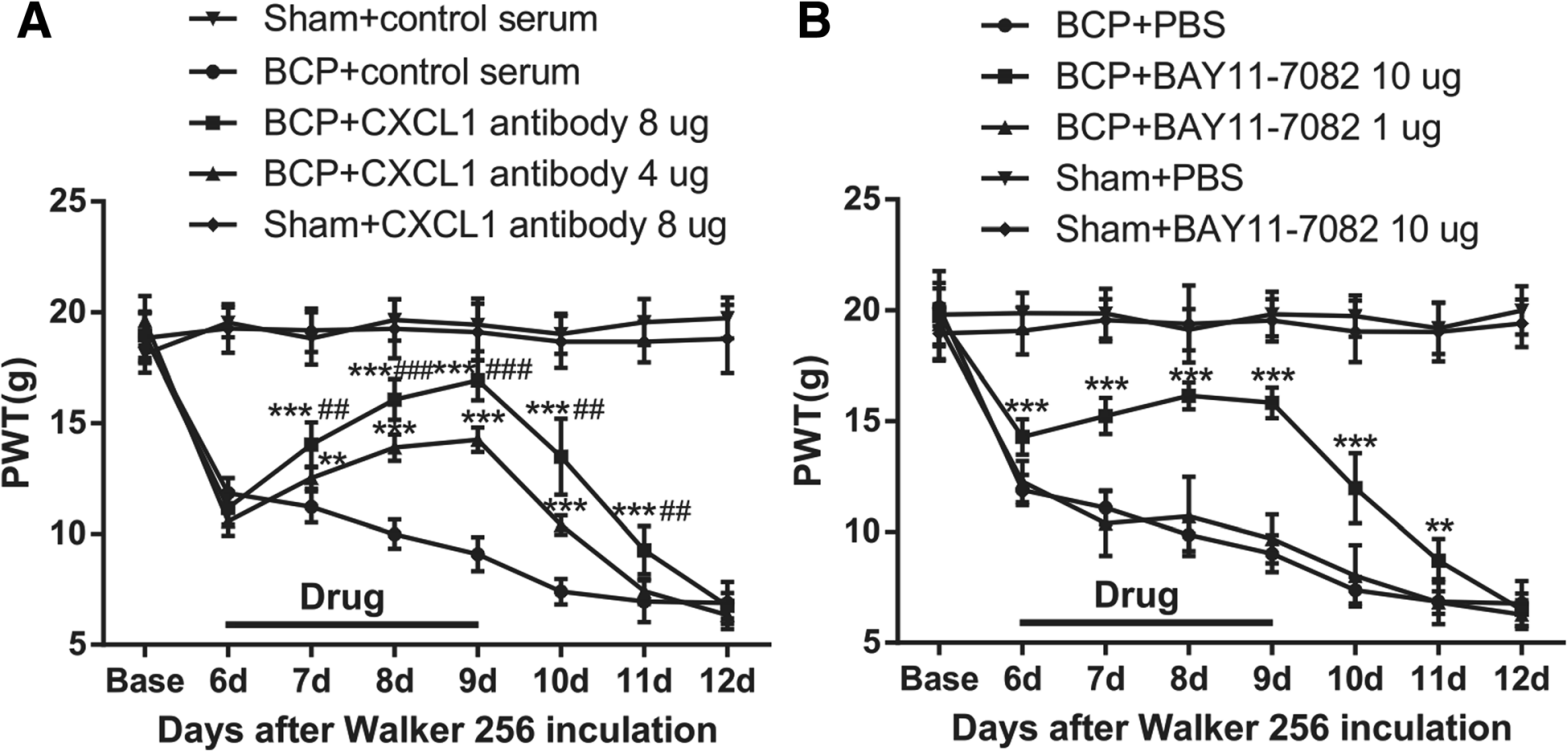
Repeated CXCL1 neutralizing antibody and NFκB inhibitor treatment attenuates mechanical allodynia caused by BCP. **a** BCP induced significant mechanical allodynia as shown by von-Frey tests. Injection of 8 μg/0.5ul and 4 μg/0.5ul CXCL1 neutralizing antibody showed an effective and reliable anti-allodynic effect in a dose-dependent manner on BCP. Drugs were given once a day from POD 6 to POD 9. *n* = 8, ***p < 0.001, **p < 0.01 compared with that of BCP + control serum group. ^###^*p* < 0.001, ^##^*p* < 0.01 compared with that of BCP + CXCL1 neutralizing antibody 4 μg group. There were eight rats in each group. **b** Repeated BAY11-7082 treatment at 1 μg showed no effect on mechanical allodynia, but 10 μg of this compound significantly attenuated mechanical allodynia (***p < 0.001, **p < 0.01 vs. BCP + PBS group; *n* = 8)

To elucidate whether suppressing vlPAG CXCL1 could inhibit pain-related behaviors following BCP, we injected intra-vlPAG into a CXCL1 neutralizing antibody (4 μg, 8 μg/0.5 μl) or control serum into the BCP rats once daily for four consecutive days (from day 6 to 9 after BCP, the pain-related behaviors are well-established and the upregulation of CXCL1 remained at a high level) and then detected the analgesic effect of CXCL1 inhibition 1 h later after micro-injection on postoperative days (POD) 6, 7, 8, 9, 10, 11, and 12, respectively. The injection of CXCL1 neutralizing antibody (8 μg/0.5 μl) into the vlPAG of sham rats showed no detectable behavioral changes relative to the baseline values. Compared with BCP + serum group, micro-administration of CXCL1 neutralizing antibody significantly elevated the PWT of the BCP rats in a dose-dependent manner (*F*_4,35_ = 862.5, ***p < 0.001, **p < 0.01 vs. BCP + serum group; ^###^*p* < 0.001, ^##^*p* < 0.01 vs. BCP + CXCL1 neutralizing antibody 4 μg group; *n* = 8, two-way repeated measures ANOVA, Fig. 5a), which effectively started on POD 7, 1 day after the beginning of drug treatment. The analgesic effect of CXCL1 neutralizing antibody (8 μg) was still observed at POD 10, 2 days after the treatment was stopped. Yet, the effect of CXCL1 neutralizing antibody (4 μg) at lower dosages lasted only for 24 h after drug withdrawal. These data suggested that CXCL1 in vlPAG was involved in BCP hypersensitivity.

We then investigated the role of pNFκB in the maintenance of BCP. We blocked NFκB pathway with an effective and specific inhibitor (BAY11-7082, 1 μg, 10 μg/0.5 μl, once daily from day 6 to 9 after BCP). The injection of BAY11-7082 (10 μg/0.5 μl) into the vlPAG of sham rats showed no detectable behavioral changes relative to the baseline values. Repeated BAY11-7082 treatment with a dose of 1 μg demonstrated no effect on mechanical allodynia, but treatment with 10 μg of this compound significantly attenuated mechanical allodynia. The effect started on POD 6, i.e., on the day of treatment initiation (*F*_4,35_ = 567.3, ***p<0.001, **p<0.01 vs. BCP + PBS group; *n* = 8, two-way repeated measures ANOVA, Fig. 5b). These data suggested that NFκB in vlPAG may be involved in BCP hypersensitivity.

### BCP induces CXCL1 upregulation in vlPAG astrocytes via NFκB pathway

**Fig. 6:**
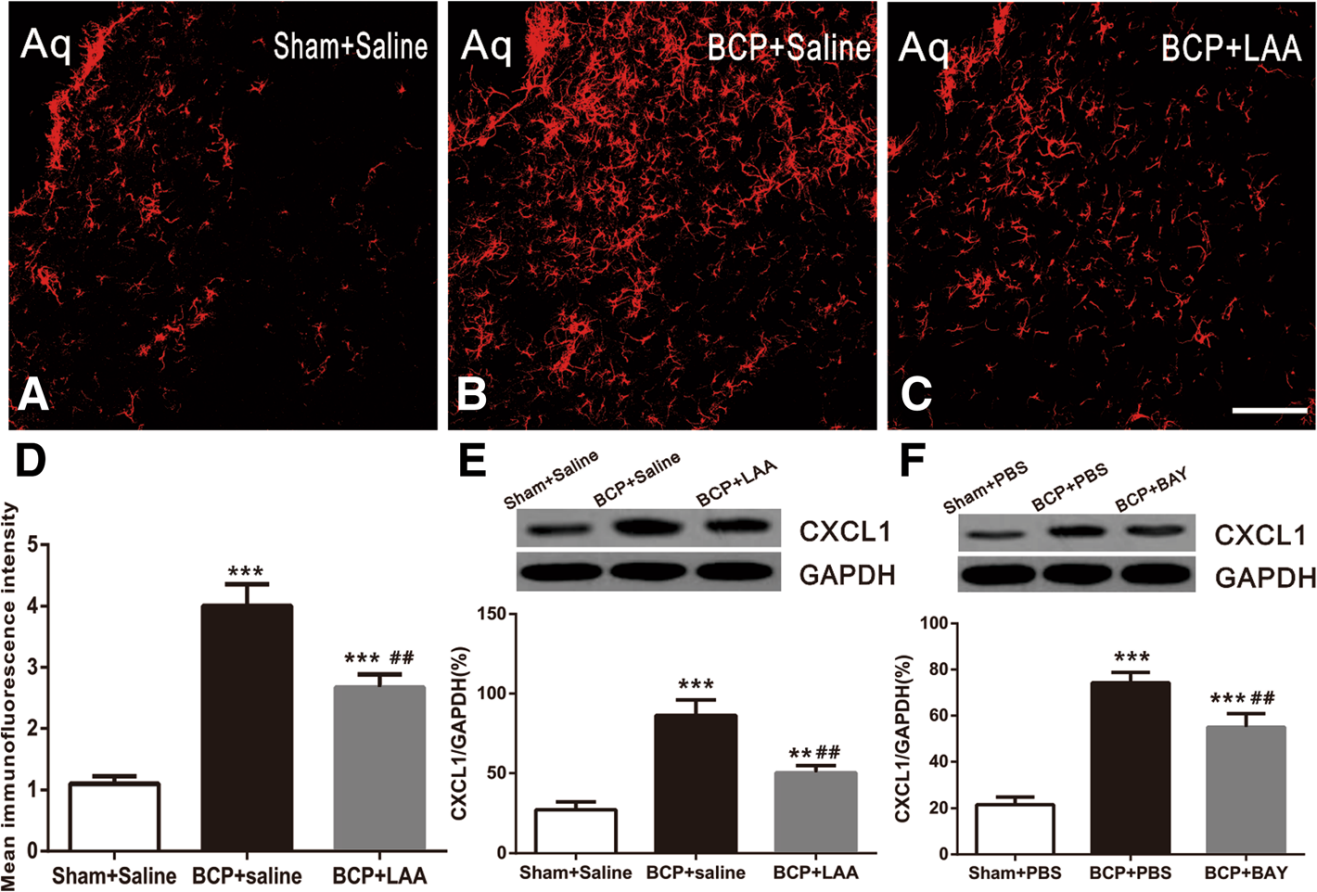
Intra-vlPAG administration of L-α-aminoadipate or BAY11-7082 reverses BCP-induced upregulation of CXCL1 protein. **a**–**c** Confocal images showed inhibitory effect of LAA on induction of astrocytic marker GFAP (red) in vlPAG after BCP. **d** Mean red immunofluorescence intensity of GFAP of (**a**) among sham, BCP, and BCP + LAA group. For control experiments, saline was used. LAA (100 nmol/0.5 μl) or saline (0.5 μl) was given once a day on POD 6, 7, 8, and 9, respectively. Tissues were collected 1 h after the last micro-injection (*n* = 4 in each group). ***p < 0.001, versus sham + saline. ^##^*p* < 0.01 versus BCP + saline. **e** Western blot and data summary showed the inhibitory effects of LAA on BCP-induced increased expression of CXCL1 protein (*n* = 4 in each group). ***p < 0.001, **p < 0.01 versus sham + saline. ^##^*p* < 0.01 versus BCP + saline. **f** Western blot and data summary showed the inhibitory effects of BAY on BCP-induced increased expression of CXCL1 protein (*n* = 4 in each group). BAY (10 μg/0.5 μl,) or PBS (for control, 0.5 μl) was given once a day on POD 6, 7, 8, and 9, respectively. Tissues were collected 1 h after the last micro-injection. ***p < 0.001 versus sham + PBS. ^##^*p* < 0.01 versus BCP + PBS. Aq, aqueduct. LAA, L-α-aminoadipate. BAY, BAY11-7082

Our recent study found that vlPAG astrocytes upregulation has an important role in maintaining the hypersensitivity of BCP. The injection of LAA (100 nmol) into the vlPAG of sham rats did not produce detectable behavioral changes relative to the baseline values (data is being published). In order to examine whether the production of CXCL1 is required for the activation of astrocytes, we micro-injected an astrocytic cytotoxin L-α-aminoadipate (LAA 100 nmol) into the BCP rats once daily for four consecutive days (from days 6 to 9 after BCP). Furthermore, the GFAP immunoreactivity and CXCL1 protein level after the last injection were detected. Compared with sham group rats, the immunofluorescence intensity of GFAP and protein expression level of CXCL1 in vlPAG were increased on day 9 in BCP rats. After repeated LAA administration, the BCP-induced GFAP immunofluorescence expression was abolished (*F*_2,9_ = 57.41, ***p < 0.001 versus sham + saline. ^##^*p* < 0.01 versus BCP + saline; *n* = 4, one way ANOVA, Fig. 6a–d). Furthermore, the BCP-induced CXCL1 protein upregulation was also inhibited when associated with decreased GFAP (*F*_2,9_ = 58.39, ***p < 0.001, **p < 0.01 versus sham + saline. ^##^*p* < 0.01 versus BCP + saline; *n* = 4, one way ANOVA, Fig. 6e). In contrast, saline did not affect GFAP immunofluorescence intensity or CXCL1 protein expression in BCP rats.

Recent studies have shown that NFκB mediated CXCL1 production in spinal astrocytes and cultured astrocytes in BCP [17]. To further demonstrate whether NFκB is the upstream of CXCL1 in vlPAG after BCP, we blocked NFκB pathway with an effective and specific inhibitor (BAY11-7082, 10 μg/0.5 μl, once daily from day 6 to 9 after BCP) and then detected CXCL1 protein level after the last injection. Compared with the control vehicle, BAY11-7082 reversed BCP-induced CXCL1 increase after four consecutive days (*F*_2,9_ = 128.4, ***p < 0.001 versus sham + PBS. ^##^*p* < 0.01 versus BCP + PBS; *n* = 4, one way ANOVA, Fig. 6f). Collectively, these findings suggested that NFκB mediated BCP-induced production of CXCL1 in activated astrocytes in vlPAG and the effect of CXCL1 was downstream to NFκB signaling.

### VlPAG injection of exogenous CXCL1 induces mechanical allodynia and activation of PAG neurons through CXCR2

**Fig. 7:**
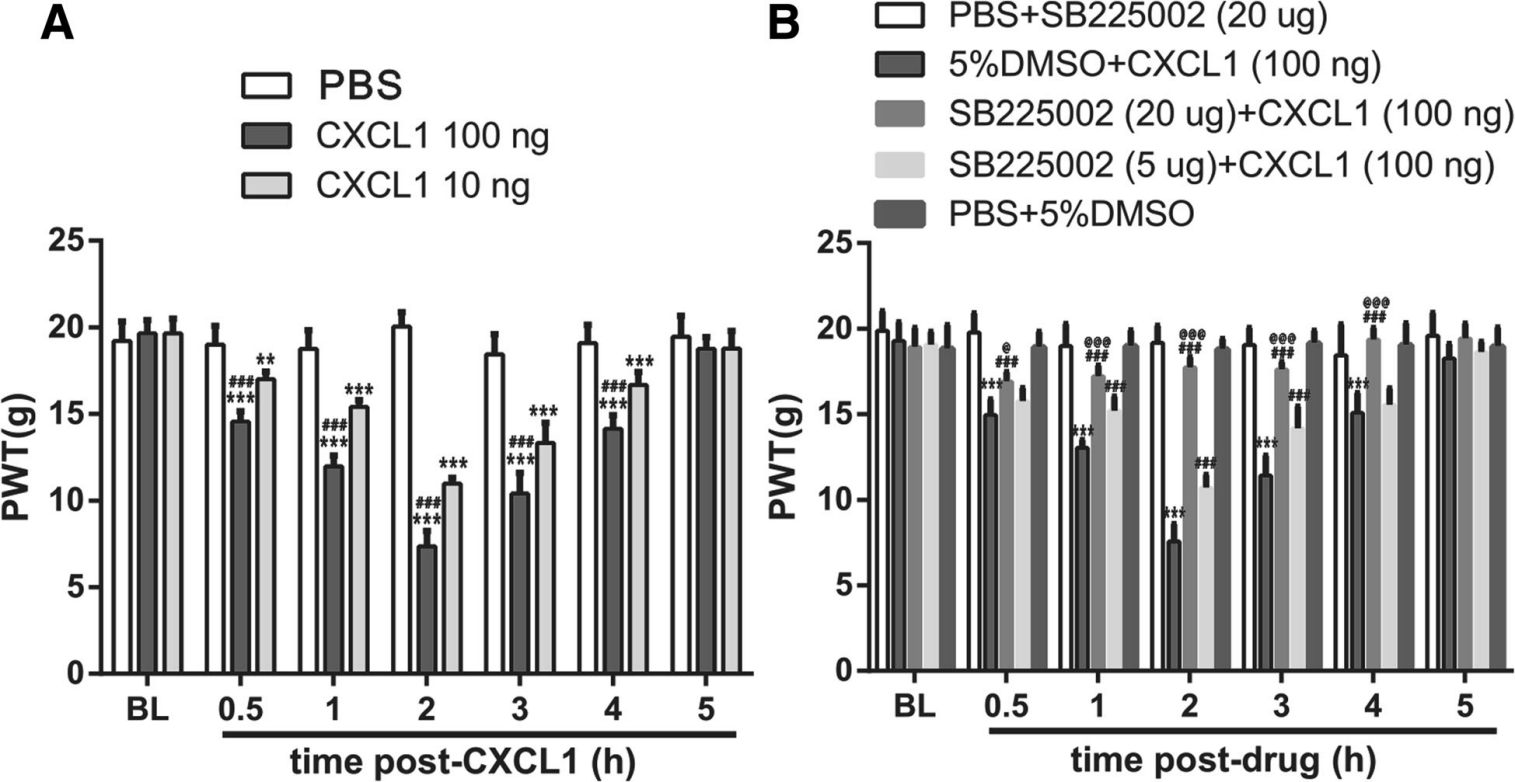
VlPAG micro-injection of CXCL1 induces mechanical allodynia via CXCR2. **a** Micro-injection of CXCL1 (10 or 100 ng) induces a dose-dependent mechanical allodynia. *n* = 8, ***p < 0.001 **p < 0.01 versus PBS; ^###^*p* < 0.001 versus CXCL1 10 ng. **b** Micro-injection of CXCR2 antagonist, SB225002, 30 min before CXCL1 vlPAG injection dose-dependently prevented CXCL1-induce mechanical allodynia. *n* = 8, ***p < 0.001 versus corresponding BL (baseline); ^###^*p* < 0.001 versus 5%DMSO + CXCL1 (100 ng); ^@@@^*p* < 0.001 ^@^*p* < 0.05 versus SB225002 (5 μg) + CXCL1 (100 ng)

We investigated whether CXCL1 was sufficient to induce pain and how it is involved in the modulation of pain hypersensitivity. We first examined the effect of direct application of CXCL1 (10 ng or 100 ng/0.5 μl) into vlPAG for nocifensive behavior in naive rats. The results showed that CXCL1 produced mechanical allodynia in a dose-dependent manner (*F*_2,21_ = 692.4, ***p<0.001 **p<0.01 vs. PBS group; ^###^*p*<0.001 vs. CXCL1 10 ng group; *n* = 8, two-way repeated measures ANOVA, Fig. 7a). The PWT was decreased at 30 min, still decreased at 2 h, and recovered after 5 h by CXCL1 100 ng.

CXCR2 is a major receptor of CXCL1 [19, 30]. To test whether CXCL1-induced pain hypersensitivity was mediated by CXCR2, we micro-injected SB225002, a potent and selective CXCR2 antagonist, into vlPAG 30 min before CXCL1 injection and tested for mechanical allodynia. Pretreatment with SB225002 (5 μg or 20 μg/0.5 μl) reversed CXCL1-induced mechanical hyperalgesia in a dose-dependent manner (*F*_6,245_ = 79.3, ***p < 0.001 versus corresponding BL (baseline); *F*_2,245_ = 136.2, ^###^*p* < 0.001 versus 5%DMSO + CXCL1 (100 ng); ^@@@^*p* < 0.001 ^@^*p* < 0.05 versus SB225002 (5 μg) + CXCL1 (100 ng); *n* = 8, three-way repeated measures ANOVA, Fig. 7b). These results are consistent with the role for CXCL1 in the descending facilitation of nociception and hyperalgesia.

### BCP induces CXCR2 upregulation in vlPAG neurons

**Fig. 8:**
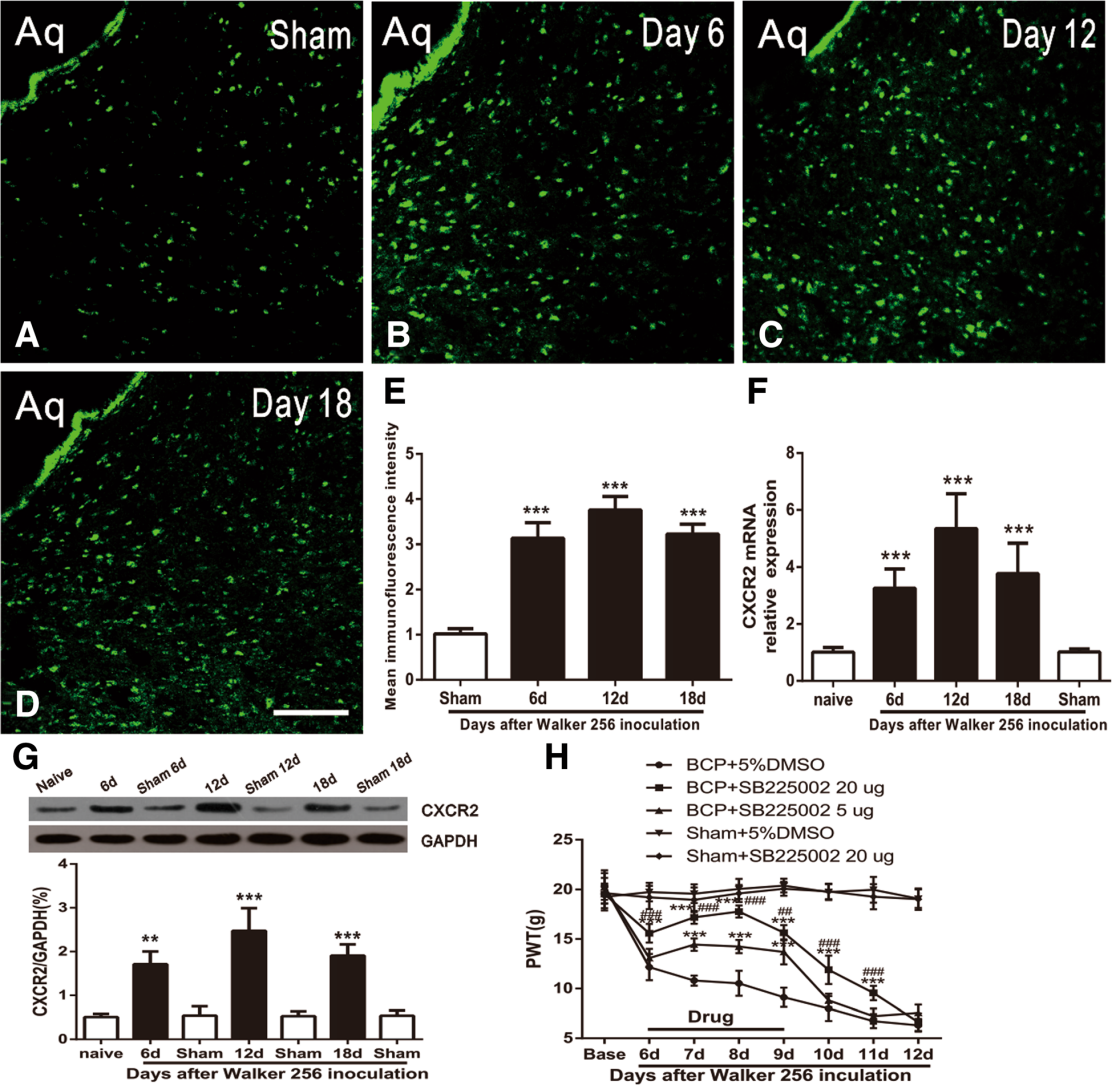
BCP increases CXCR2 mRNA and protein expression in the vlPAG. **a** Expression levels of CXCR2 in the vlPAG in sham group. **b**–**d** Upregulation of CXCR2 in vlPAG on POD 6, 12, and 18. Scale bars: 100 μm; Aq, aqueduct. **e** The statistical analysis of CXCR2 intensity confirmed the increase of CXCR2 expression in vlPAG after BCP. ***p < 0.001 vs. sham group. *n* = 4 rat per group. **f** Real-time PCR results show the increase of CXCR2 mRNA expression in the vlPAG. CXCR2 mRNA was increased from 6 days to 18 days after inoculation. ***p < 0.001 vs. naive group. *n* = 4 rat per group. **g** Western blot further confirmed a parallel and significant increase in vlPAG at 6, 12, and 18 days in BCP. ***p < 0.001, **p < 0.01 vs. naive group. *n* = 4 rat per group. **h** SB225002 attenuated Walker 256 cell inoculation-induced mechanical allodynia in a dose-dependent manner. ***p<0.001 vs. BCP + 5%DMSO group; ^###^*p*<0.001, ^##^*p*<0.01 vs. BCP + SB225002 5 μg group; *n* = 8

We further investigated CXCR2 expression and distribution in vlPAG in BCP. Immunostaining showed a low expression of CXCR2 in the sham group rats (Fig. 8a) and an increased expression in BCP rats at POD 6, 12, and 18 (Fig. 8b–d). Statistical analysis of CXCR2 intensity confirmed the increase of CXCR2 expression in vlPAG after BCP (*F*_3,12_ = 86.13, ***p<0.001 vs. sham group; *n* = 4, one way ANOVA, Fig. 8e). These findings were additionally confirmed by RT-PCR. The RT-PCR results showed that CXCR2 mRNA was increased for 6 days and maintained for more than 18 days (*F*_4,15_ = 21.59, ***p<0.001 vs. naive group; *n* = 4, one way ANOVA, Fig. 8f). Western blot results further revealed that CXCR2 expression was gradually increased from 6 days to 18 days (*F*_6,21_ = 39.48, ***p<0.001, **p<0.01 vs. naive group; *n* = 4, one way ANOVA, Fig. 8g).

To investigate the role of CXCR2 in vlPAG after BCP, CXCR2 antagonist, SB225002 (5 μg and 20 μg, once daily from day 6 to 9 after BCP) was micro-administered. The SB225002 at a dose of 5 μg gradually showed an ameliorative efficacy from days 7–9, but 20 μg significantly reduced mechanical allodynia from day 6 to day 11 in a dose-dependent manner (*F*_4,35_ = 1039, ***p<0.001 vs. BCP + 5%DMSO group; ^###^*p*<0.001, ^##^*p*<0.01 vs. BCP + SB225002 5 μg group; *n* = 8, two-way repeated measures ANOVA, Fig. 8h), suggesting the involvement of CXCR2 in the vlPAG after BCP.

**Fig. 9:**
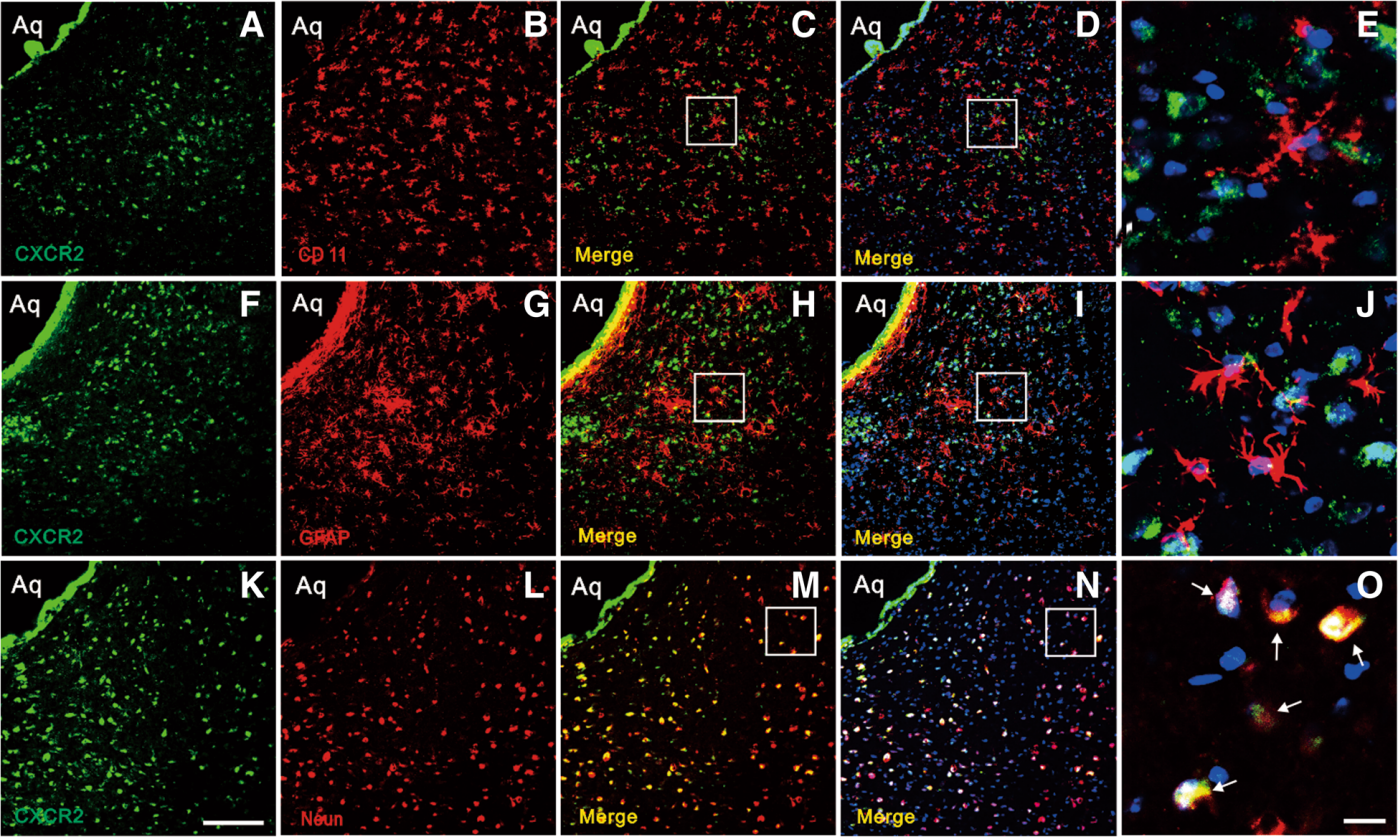
Cellular localization of CXCR2 expression in vlPAG after BCP. Immunofluorescence triple staining of CXCR2 (**a**, **f**, **k**), GFAP (**g**), Neun (**l**), CD11 (**b**), and DAPI in vlPAG at day 6 after BCP show that all glial cells and neurons express DAPI + cells (**d**, **i**, **n**). Scale bar in (**k**): 100 μm. Immunofluorescence triple staining of high-magnification images, indicated in the white boxes of (**c**, **h**, **m**, **d**, **i**, **n**), mainly mainly co-localized with Neun (**o**), but not with astrocyte (**j**) and microglia (**e**). Scale bar in (**o**): 20 μm. Aq, aqueduct. *n* = 4

To further define the cellular localization of CXCR2 in vlPAG, we performed triple staining of CXCR2 with three different cell markers (Neun, GFAP, and CD11) and DAPI. Immunofluorescence triple staining showed that CXCR2 was increased in vlPAG from day 6 after BCP (Fig. 9a, f, k) and mainly co-localized with Neun (Fig. 9k–o), but not with astrocytes (Fig. 9f–j) and microglia (Fig. 9a–e) in BCP rats.

## Discussion

Among the inflammatory chemokines, the role of CXCL1 in pain hypersensitivity has attracted considerable attention [14–18]. Herein, we showed that CXCL1 in PAG as a substantial component of the descending pain modulatory network and was upregulated in the model of BCP. Firstly, BCP induced slowly (6 days) but persistently (18 days) upregulated CXCL1 in vlPAG astrocytes, which was dependent on the NFκB pathway. Microinjection of CXCL1 neutralizing antibody attenuated inoculation-induced BCP hypersensitivity. Secondly, NFκB was activated in vlPAG astrocytes and neurons after inoculation of Walker 256 cells. Inhibition of NFκB not only alleviated BCP, but also decreased CXCL1 upregulation in vlPAG. Thirdly, CXCR2, which is the major receptor of CXCL1 was increased in vlPAG neurons. Direct injection of CXCL1 into vlPAG induced dose-dependent hyperalgesia, but was prevented by pretreatment with SB225002. Microinjection of CXCR2 antagonist attenuated BCP. These results suggested that the NFκB-dependent astrocytic CXCL1 and neuron CXCR2 signaling cascades play a significant role in astroglial-cytokine-neuron interactions and in descending the facilitation of BCP.

### CXCL1 upregulation in activated astrocytes in vlPAG and the involvement in BCP

CXCL1 is one of the major chemoattractants for neutrophils [31] and is known as cytokine-induced neutrophil chemoattractant type-1 (CINC-1) in rats [32]. To date, several evidences in rats/mice experiments showed that CXCL1 was expressed at key locations [especially in the DRG and spinal dorsal horn (SC)] of the pain transmission pathway in pathological pain states. In the DRG level, CXCL1 expression was increased after 3 days but not at 7 days after model establishment of neuropathic pain [33] and localized inflammatory irritation [34]. In addition, CFA induced rapid increase of CXCL1 mRNA and protein in the DRG [35]. In the SC level, CXCL1 mRNA and protein levels are increased after nerve injury, inflammation, and tumor cell inoculation [14, 16, 17]. In the supraspinal level, CXCL1 secreted by astrocytes in the brain played a significant role in cerebral endothelial activation and leukocyte recruitment during neuroinflammation [36]. Our study showed that BCP induced a slow (6 days) but persistent (18 days) CXCL1 increase in the vlPAG, indicating that CXCL1 plays an essential role in BCP at the supraspinal level.

Furthermore, it was previously reported that CXCL1 is expressed intensely in spinal astrocytes, but not in the microglia and neurons in the pathological painstates [16, 17]. Neuronal injury and intracerebroventricular administration of endothelin-1 induced astrocytic CINC-1 production [37, 38]. CXCL1 is also expressed in the hypertrophic astrocytes after multiple sclerotic lesions in humans [39], while Johnson et al. [40] reported that CXCL1 was expressed in the brain neurons and endothelial cells after soman-induced status epilepticus in rats. Similar to the case with the spinal cord, we observed that CXCL1 in vlPAG was present in astrocytes, but not in neurons or microglia. This finding not only provides further evidence about the action of PAG glial cells on BCP (paper is being published), but also showed that CXCL1 was upregulated in vlPAG along with astrocyte activation by the development of BCP in rats for the first time.

It has been clearly proved that astrocytes in the central nervous system (CNS) play an essential role in several neuronal functioning aspects, including pain processing [12, 41, 42]. Astrocytes are identified as sources of algogenic substances, because large amount of evidences demonstrated that activated astrocytes can release pro-inflammatory cytokines (such as IL-1β, IL-6, and TNF-α) and chemokines (such as MCP1, CXCL1, and CXCL12) in the CNS to enhance and prolong the pain processing [26, 43, 44]. Shen et al. showed that fluorocitrate, a general glial metabolic inhibitor, exerted an inhibitory effect on the induction of astrocytic marker GFAP and blockade of CXCL12 induction in bone cancer states [45]. Consistent with this study, we showed that the BCP-induced CXCL1 protein and GFAP expression upregulation were also inhibited after repeated micro-administration of LAA. These data further indicated that chemokine CXCL1 was also released from the activated astrocytes.

Other chemokines, such as MCP1, are recently found to be involved in descending facilitation of neuropathic pain. Neutralizing CCL2 with an anti-CCL2 antibody into the rostral ventromedial medulla after spinal nerve ligation significantly attenuated the established thermal and mechanical hypersensitivity [46]. In our study, continuous intra-vlPAG of CXCL1 neutralizing antibody after inoculation significantly elevated the PWT of BCP rats in a dose-dependent manner. These data suggested that CXCL1 in vlPAG is one of the astrogliotransmitters that regulate the descending facilitation of nociception and hyperalgesia.

### NFκB mediates CXCL1 upregulation and descending pain facilitation

NFκB, a transcription factor, is involved in the expression of genes related to the inflammatory process, including regulated CXCL1 expression [47]. NFкB has been found to regulate CXCL1 transcription in Hs294T malignant cells [48]. Xu et al. reported that NFκB is involved in CXCL1 production in spinal astrocytes and cultured astrocytes [17]. Evidence confirms that the phosphorylation of NFκB (p-NFκB) is related to the development and maintenance of neuropathic pain and BCP. Inhibition of p-NFκB in the spinal level by BAY11-7082 partly prevented the development of neuropathic pain and attenuated the established BCP [26, 49].

The PAG of the brainstem are considered as the essential structures of the endogenous pain modulatory system that alter the spinal cord processing of sensory input [50]. Despite of a growing body of evidence indicating a role of PAG in descending pain modulation [12, 51], the precise underlying cellular and molecular mechanisms involved in descending facilitation and inhibition of the dorsal horn remains elusive. The midbrain vlPAG has been shown to be a principal component mediating pain modulation [3, 5].

To check whether NFκB pathway in vlPAG was involved in BCP hypersensitivity, Western blot and RT-PCR results showed that the expression of pNFκB was gradually increased from 6 days to 18 days. We also showed that NFκB inhibitor reduced BCP and CXCL1 expression in vlPAG. These results indicated CXCL1 as an important downstream of NFκB activation in mediating the process of BCP and supported the role of NFκB in the maintenance of BCP via CXCL1 production in vlPAG.

Our data further showed that pNFκB was predominantly expressed in astrocytes and neurons, but not in microglia of vlPAG. Consistently, pNFκB expression in astrocytes was found in the spinal cord following spinal nerve injury [52] and inoculation of RM-1 cells into the femur [17]. pNFκB expression was also observed in the medullary dorsal horn in rats with trigeminal neuropathic pain [53]. However, Bethea et al. reported activated NFκB in spinal microglia, endothelial cells, and neurons after spinal cord injury [54]. Our laboratory recently reported predominant expression of pNFκB in neurons [26]. The discrepency of cellular distribution of pNFκB may be due to the use of different animal species or different antibodies or different action sites, which needs to be further explored in future.

### CXCL1-CXCR2 signaling cascades play a role in glia-neuron interactions

The role of CXCL1 is dependent on its primary receptor CXCR2 [19, 55]. Accumulating evidences suggest that glial-neuron interactions in the dorsal horn contributed to the central sensitization under pathological conditions [9, 56, 57]. The contribution of glial-neuron interactions in the supraspinal pain-modulatory circuitry to persistent pain has also been recognized [46, 58]. Our convergent results indicated that intra-vlPAG of CXCL1 into vlPAG promoted pain hypersensitivity. In agreement with the upregulation of CXCL1 and CXCR2, antagonism of CXCL1-CXCR2 was attenuated and CXCL1 agonist stimulation enhanced the nocifensive behavior.

CXCR2 was highly expressed by subsets of projection neurons in various regions of the spinal cord and brain [16, 59]. The CXCR2 receptor has been detected on oligodendrocyte progenitors [60, 61] and microglia [62] in the brain. In our study, immunostaining showed that the tumor cell inoculation increased CXCR2 expression in vlPAG neurons. Both Western blot and RT-PCR confirmed that BCP increased CXCR2 expression in vlPAG. Behavioral results further demonstrated that CXCR2 antagonist attenuated BCP hypersensitivity after tumor cell inoculation in a dose-dependent manner, suggesting that enhanced neuronal CXCR2 also occurs in the supraspinal pain modulatory circuitry. The respective expression of CXCL1 and CXCR2 in astrocytes and neurons in vlPAG may be involved in astroglial-neuronal interaction.

## Conclusions

Our present study suggests that inoculation of Walker 256 mammary gland carcinoma cells into the rat tibia induced bone destruction and pain hypersensitivity. In association with these changes, rats showed a prominent expression of CXCL1/CXCR2 in vlPAG, which in turn may activate astrocytes and neurons, respectively. Moreover, NFκB was involved in the production of CXCL1 in vlPAG astrocytes. Our data suggests that crosstalk between NFκB-dependent astrocytic CXCL1 and neuron CXCR2 plays a role in descending pain facilitation. Signal coupling between CXCL1 and CXCR2 receptors may lead to increased descending output from pain modulatory network, facilitating spinal nociceptive transmission and intensifying the perceived pain, and remains to be the focus of our future research.

## Additional file

Additional file 1: **Figure S1.** A representative photomicrograph of a coronal slice at the level of the vlPAG stained with red fluorescent stain. Image shows the injection site in vlPAG. Coronal brainstem sections were stained with red fluorescent stain. Arrow indicates the cannulation track and circle shows the site of injection. Scale bars = 100 um; Aq, aqueduct. (TIF 5263 kb)

## Abbreviations

BCP: Bone cancer pain
CXCL1: Chemokine C-X-C motif ligand 1
CXCR2: Chemokine CXC receptor 2
GAPDH: Glyceraldehyde-3-phosphate dehydrogenase
GFAP: Glial fibrillary acidic protein
GRO: Growth-related oncogene
KC: Keratinocyte-derived chemokines
NFкB: Nuclear factor kappa B
PAG: Periaqueductal gray
PWT: Paw withdrawal threshold
RVM: Rostral ventromedial medulla
vlPAG: Ventrolateral periaqueductal gray
